# Oxygen storage module with physisorption technology for closed-circuit respirators

**DOI:** 10.1088/1757-899x/755/1/012106

**Published:** 2020-03

**Authors:** A M Swanger, J E Fesmire, R Fernando

**Affiliations:** 1 NASA Kennedy Space Center, Cryogenics Test Laboratory, Kennedy Space Center, FL 32899 USA; 2 National Institute for Occupational Safety and Health, Pittsburgh, PA 15236 USA

## Abstract

The new Cryogenic Flux Capacitor (CFC) technology employs nano-porous aerogel composites to store large quantities of fluid molecules in a physisorbed solid-state condition at moderate pressures and cryogenic temperatures. By its design architecture, a CFC device can be “charged” and “discharged” quickly and on demand according to standby/usage requirements. One of three main application areas is the CFC-Life for breathing air or oxygen supply to meet new demands in life support systems. Through the Liquid Oxygen Storage Module (LOXSM) Project, the National Institute for Occupational Safety and Health, and Cryogenics Test Laboratory have partnered to test the feasibility of applying the CFC technology to Closed-Circuit Escape Respirators (CCER), or respirators operating on the closed-circuit principle in general. The envisioned Cryogenic Oxygen Storage Module (COSM) is an innovative concept to store oxygen in solid-state form, according to physisorption processes at any cryogenic temperature, and deliver it as a gas using the CFC as the core storage element. Gaseous oxygen would be admitted into the breathing loop of the CCER by introducing heat into the storage module. Potentially replacing the gaseous or chemical based oxygen supply used in today’s closed-circuit respirators, the COSM is a high capacity, conformal, small-size solution for future life support equipment of all kinds. In particular, are the CCER devices that must to be carried on the person, ready to be quickly deployed and used for escape in an emergency. Initial test data for physisorption of oxygen in aerogel materials and CFC core modules are presented. The basic operational parameters for charging and discharging are summarized through prototype testing of the cryogenic oxygen storage module.

## 1. Introduction

Storage of oxygen (O_2_) for breathing apparatuses is typically done in one of two ways: as low pressure, cryogenic liquid (LO_2_) or as high-pressure gas (GO_2_). Cryogenic liquid tanks use vacuum-jacketing, low heat-leak supports, and carefully designed piping connections to enable storage of liquid with reasonably low boil-off losses. Such tanks cannot be made in conformal shapes and tend to be complex and heavy. Conversely, high pressure gas storage cylinders are not affected by tank orientation, operate at ambient temperature, and hence are much less complicated pieces of equipment. However, gas storage vessels, even at extreme pressures up to 700 bar, have limited capacity due to gas compressibility factors, especially at higher pressures, and require highly specialized components, materials, and cleaning specifications related to oxygen sensitivity. These two options are typically traded depending on the system requirements, but few practical options exist that provide all the benefits while limiting the downfalls.

Closed-circuit respirators (CCRs) are breathing apparatus that function in a re-circulatory mode whereby the user breathes the life support gas in a closed loop. Exhaled carbon dioxide (CO_2_) is removed by an absorber and oxygen is added to the system according to the user’s metabolic needs. Typical CCR designs use chemicals such as calcium hydroxide and lithium hydroxide as CO_2_ absorbents. Gaseous oxygen stored in high pressure cylinders or oxygen generating chemicals such as potassium superoxide serve as the oxygen source. Closed-circuit escape respirators (CCERs) are smaller versions of CCRs used for escape purposes and usually carried by the person at all times, ready to be used in an emergency. Next Generation CCERs need to be designed for comfortable and continuous wear by persons during their daily work routines, as well as for function as an escape breathing apparatus in the event of an emergency. Therefore, due consideration should be given in the design to minimize size and weight of these units. For use by the workforce in the United States, CCER designs must meet the applicable provisions within the Code of Federal Regulations Title 42, Part 84, including Subpart O. For example, the gaseous oxygen capacity (at standard conditions) of the units for shipboard escape, as required by the U.S. Navy is Cap 1–25 liters GO_2,_ and for mine escape it is Cap 3–81 liters GO_2_, as defined in Subpart O, to meet mining regulatory requirements [1].

Using physisorption technology combined with cryogenic refrigeration, an alternative method of fluid storage is being developed for oxygen storage. Net storage density is higher than high pressure gas and on par with liquid density but without the limitations of these storage methods. The physisorption technology is based on a Cryogenic Flux Capacitor (CFC) [2] system that includes core module for oxygen charging at any cryogenic temperature and then discharging upon demand for GO_2_ supply.

Using CFC-based oxygen storage modules, the goal is to reduce the overall size and weight of CCERs (and CCRs in general), for the same oxygen capacity as a conventional apparatus that uses chemical or gaseous oxygen. Further advantages are that it is a high-capacity, small-size storage module that can be formed into conformal shapes for a compact respirator design. The new cryogenic oxygen storage module could potentially reduce the size of the CO_2_ absorber, or even eliminate the need for it, by sequestering CO_2_ from the exhaled air. As part of the National Institute for Occupational Safety and Health (NIOSH) funded LOXSM Project to develop new oxygen breathing equipment, prototypes of the CFC-based modules have been produced and tested by the Cryogenics Test Laboratory at NASA’s Kennedy Space Center. The prototype units are designed with integral heat exchange capability to allow quick oxygen charging and controlled discharging within the specified range of flow rate. Future plans are to incorporate these modules into closed-circuit respirator test platforms for extensive testing. Key tests planned are oxygen static retention time, dosage control, and performance tests as detailed in regulation 42 CFR 84-O. The status of prototype module development and testing is reported here.

## 2. Cryogenic Flux Capacitor for storage of oxygen

The Cryogenic Flux Capacitor is an energy-based device for the storage (charging) and un-storage (discharging) of fluids in a practical way. The stored energy in this case is represented by fluid molecules accumulated in a solid-state manner. Solid-state in this context means that the fluid atoms or molecules are physically bonded within the pores of a meso-porous or nano-porous storage media—in other words, the fluid charged within a CFC is phase-less in the classical sense; however, the fluid is always discharged in its gas phase. This process of bonding or debonding is governed by principles of physical adsorption (physisorption) and thermodynamics. A thorough investigation into the physics and mechanisms of physisorption can be found in Boer [3]; but in general, storage capacity increases with decreasing temperature and increasing pressure.

**Figure 1.**
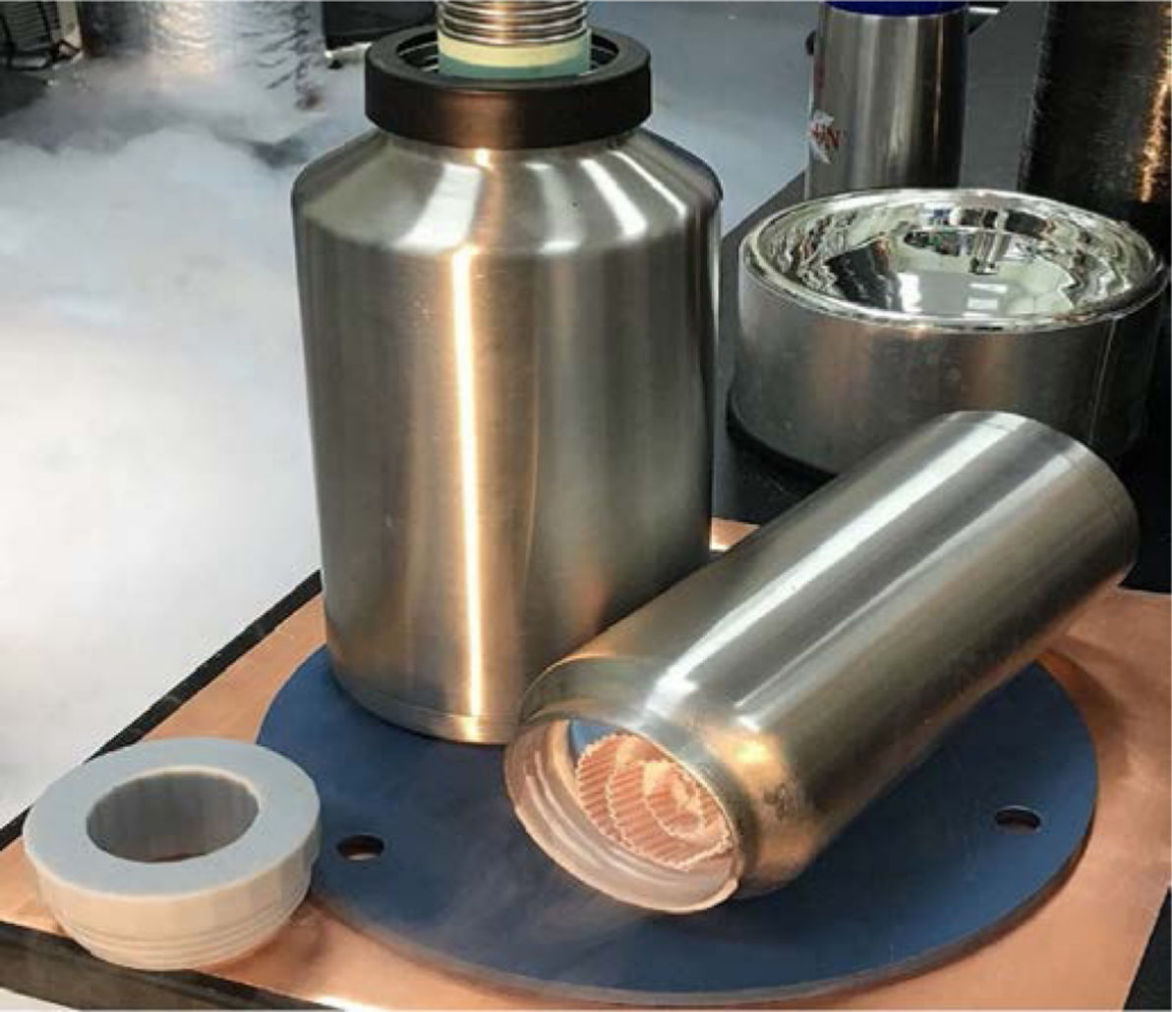
Prototype CFC core with vacuum-jacket

In a CFC-based oxygen storage module, the device can be charged by exposing the CFC core directly to liquid oxygen, or via a gaseous O_2_ supply in conjunction focused refrigeration power to the core. Problems with liquid behavior such as sloshing or liquid level management are avoided because the fluid is stored in a physisorbed state that is not liquid, no matter the density or temperature. Due to the cryogenic temperatures, the core must be thermally protected from the ambient environment to a degree commensurate with the desired dormancy for the application. Figure 1 depicts a charged CFC core prototype contained within a vacuum-jacketed vessel.

## 3. Requirements for oxygen respirator

The main requirements for the CCER (per CFR 42 special case, Subpart O, for escape applications) are listed as follows:

- Flow: 1.35 liter per minute (lpm) average flow rate or 81 liters of oxygen gas supplied in one hour (minimum); which corresponds to a liquid volume equivalent (LVE) of approximately 102 cm^3^ of liquid O_2_.
- With a O_2_ gas density at standard temperature and pressure (STP) of 1.429 g/liter, the equivalent mass flow rate is 1.93 g/minute or a total mass of 116 g over a 1 hour period
- With a liquid expansion ratio of 860, the LO2 equivalent is 94.2 milliliters
- The 1.35 lpm oxygen flow corresponds to an exhalation CO2 flow of 1.15 lpm. The respiratory coefficient is therefore 1.15/1.35 = 0.8 to 0.9 range on average.
- Maximum and minimum oxygen flow rates are: 3.00 lpm and 0.5 lpm, respectively.
- Duty cycle: respirator has to be available to work during a 10-hour shift (9-hour standby time)
- Charging: There will be a charging rack for multiple respirators.
- Standby: standby time depends on the heat leakage rate.

Three chief design problems for the module are summarized as follows: 1) standby (ambient heat leak), 2) capacity for one hour at a nominal withdrawal rate of 1.35 lpm oxygen, and 3) regulation of demand from 0.5 to 3.0 lpm oxygen. In the standby mode the module must be as thermally isolated as possible, but in the discharge mode the heat exchange with the ambient process is needed. As the CO_2_ scrubber material (e.g. lithium hydroxide) generates a lot of heat, future module designs may be able to take advantage of this heat to drive the discharge flow rate. The maximum inhalation temperature allowed for the user is 43 °C. The scrubber material itself can get up to 70°C or higher and ice or Phase Change Materials (PCM) are often used for cooling for the longer duration CCR units.

Calculations show that a CFC charged using a supply of normal boiling point LO_2_, roughly 40-mm diameter by 100-mm length has adequate storage capacity to provide the one-hour minimum of breathable oxygen at 1.35 liters per minute to a CCER per 42 CFR 84 Subpart O requirements. Additional capacity must be added for standby or dormancy requirements up to nine hours to cover a full 10-hour work shift. Prototype modules would be designed around such a CFC, and provide the appropriate thermal protection/control, pressure containment, and flow metering. It is envisioned that this cryogenic oxygen storage module (per this LOXSM project) be part of the oxygen delivery system in future closed-circuit escape respirators (CCERs).

From extensive testing of the aerogel composite materials in different fluids at cryogenic temperatures, the main design parameters can be established for a given material and fluid combination [4]. For oxygen in Cryogel material (average bulk density of 0.167 g/cm^3^), the average mass ratio (MR) is approximately 7.1 while the average volume ratio (VR) is approximately 1.03. For example, storing 116 g of oxygen requires 16.3 g of Cryogel, or about 100 cm^3^ in volume.

## 4. Heat exchange process and design concepts

To liberate (or “un-store”) the cryo-adsorbed molecules within the core of the CFC-based module, a heat source is required. Heating can be provided by a solid conduction path from the ambient environment and/or electrical heater, the exhaled breathing air, or the exothermic reaction of the associated CO_2_ scrubber material. Or the heat can be supplied from any combination of these three for optimum balancing effect to meet demand cycles from steady-state to high flow.

Most of the prior development work for the CFC technology was focused on the use of either embedded electrical heater which brings potential safety issues for an oxygen breathing apparatus.

Therefore, several options were considered for heat supply or heat exchange with the ambient:

1. Solid conduction system from external thermal mass via thermal switch
2. Tube-in-tube system with segregated pressure boundaries (i.e. no mixing between exhaled breath and discharged oxygen)
3. Flow-through system with segregated pressure boundaries
4. Flow-through system with mixed fluids (open system)

After consideration, the most promising design concepts to further explore were determined to be those that incorporate the human breath for heating in a combination of mixed and open boundaries. Conduction heating from the ambient environment is still a possibility to augment the process, but use of the heat available in the human breath is the desired method for delivering the necessary heat to drive the desorption process. In this way, the heat flow can potentially be made commensurate with the breathing rate (at rest or peak) such that a fully passive-acting heat exchanger system would be feasible.

**Figure 2.**
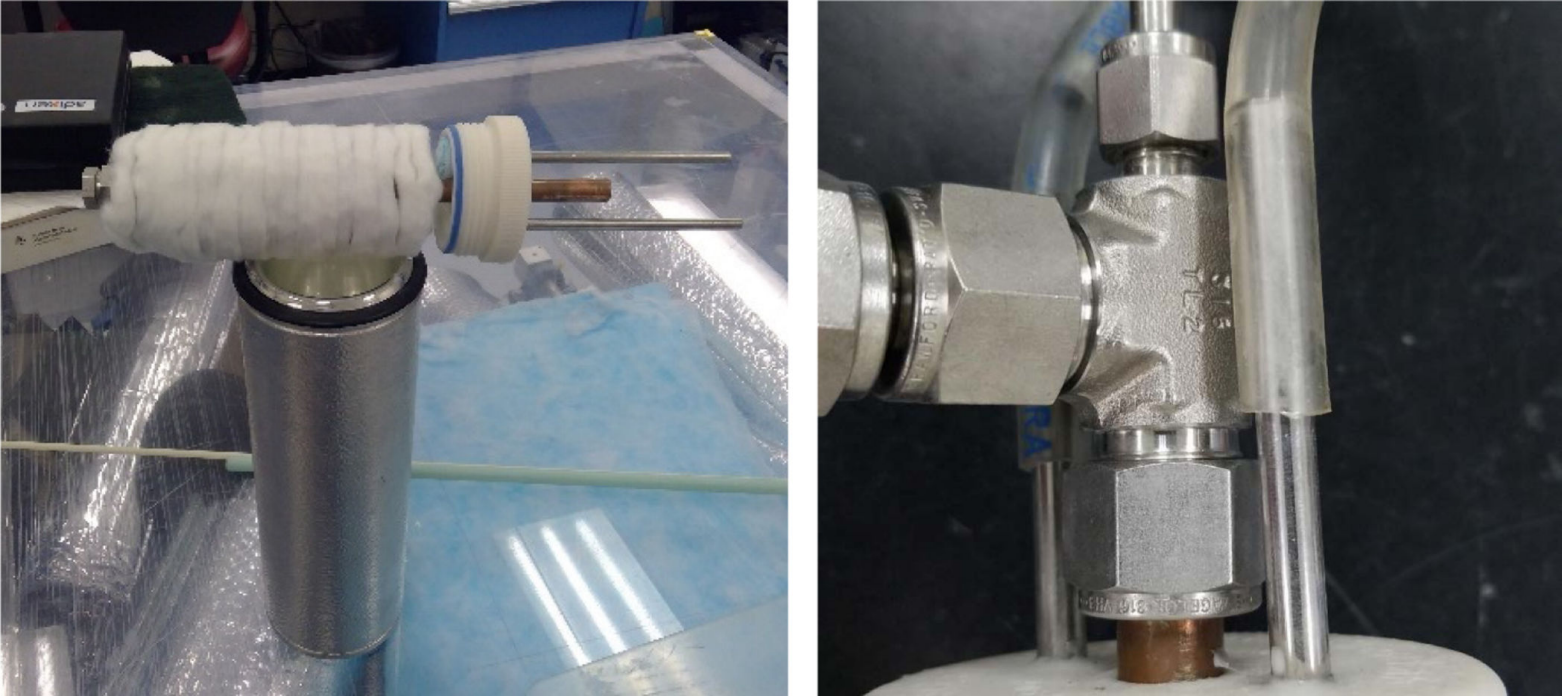
Prototype-1 with flask (left); detail of tube-in-tube counter-flow heat exchanger (right)

A CFC concept using a counter-flow (tube-in-tube) heat exchanger was designed with a parallel-plate stack-up arrangement of the aerogel adsorption media as shown in figure 2. A functioning prototype to act as a cryogenic capacitor for use in an emergency breathing air apparatus was then fabricated in this manner. This prototype module (Prototype-1) was designed to store adsorbed fluid within a stack-up of 10-mm aerogel composite disks (Cryogel^®^ by Aspen Aerogels, Inc.) mounted on a center column of the counter-flow heat exchanger device. The GN_2_ gas was released utilizing warm GN_2_ to simulate “breath” from a breathing apparatus.

**Figure 3.**
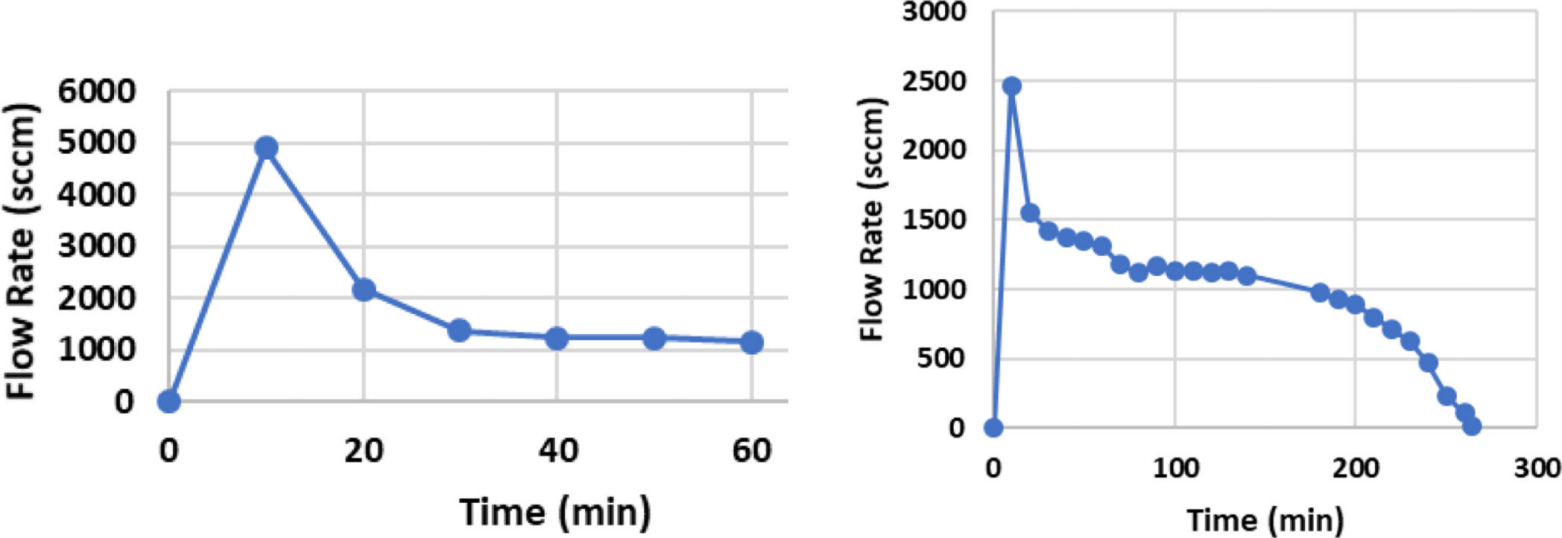
Module Prototype-1, discharge Test 1, left, and Test 2 (with precooling), right.

A one-liter capacity lab dewar flask was used to act as the vacuum-jacketed container for capacitor module. This arrangement allows for a small gap between the inner wall of the flask and the outer edge of the cap, ensuring that the vapor can properly escape. The entire assembly was placed inside the controlled environmental chamber to limit the amount of water frozen to the exposed metal components. The test procedure begins with measurement of the initial “dry” mass of the module (360g) and zeroing the scale. The module was then submerged in an LN_2_ bath and allowed to cold soak for 30 minutes. After removing the module and shaking off any excess LN_2_, the module was again placed in the flask for a final “charged” mass. Measurements were recorded every 10 minutes for one hour. For Test 1, the initial flow rate was higher due to the flask not being precooled. The total mass uptake at the start (time zero) was 260 g. As the flask reached thermal equilibrium the flow rate became stable at approximately 1,200 standard cubic centimeters per minute (sccm), or 1.20 lpm, as shown in figure 3 (left). For Test 2 the flask was precooled for 30 minutes by filling it with LN_2_. After cooldown the same process for Test 1 was repeated and the charged mass was measured to be 329 g. The flow rate results for Test 2 are given in figure 3 (right).

## 5. Modules testing with different fluids

**Figure 4.**
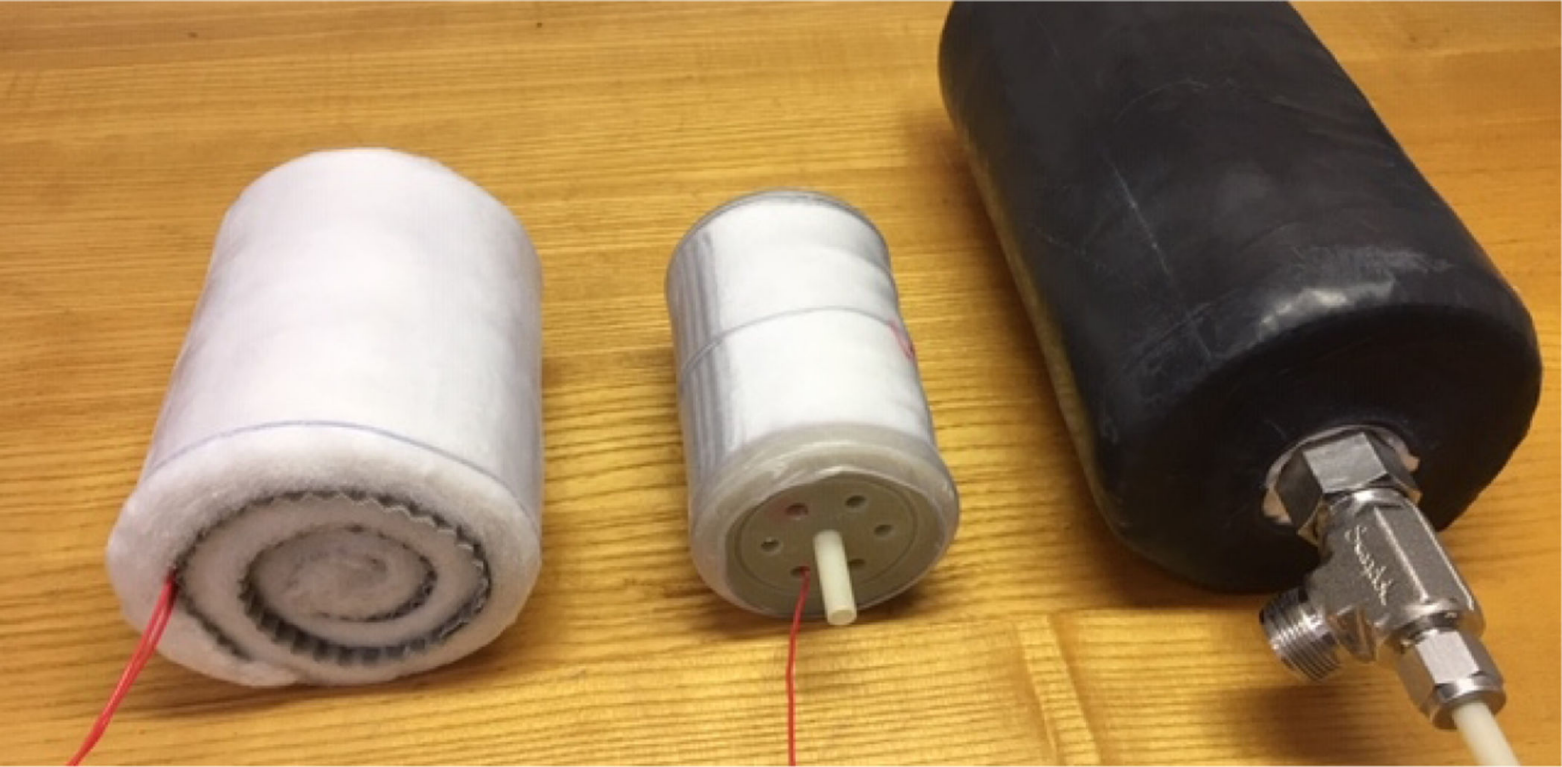
Prototype CFC core modules for cryogenic testing: Module I, Module II, and Module III (from left to right)

**Figure 5.**
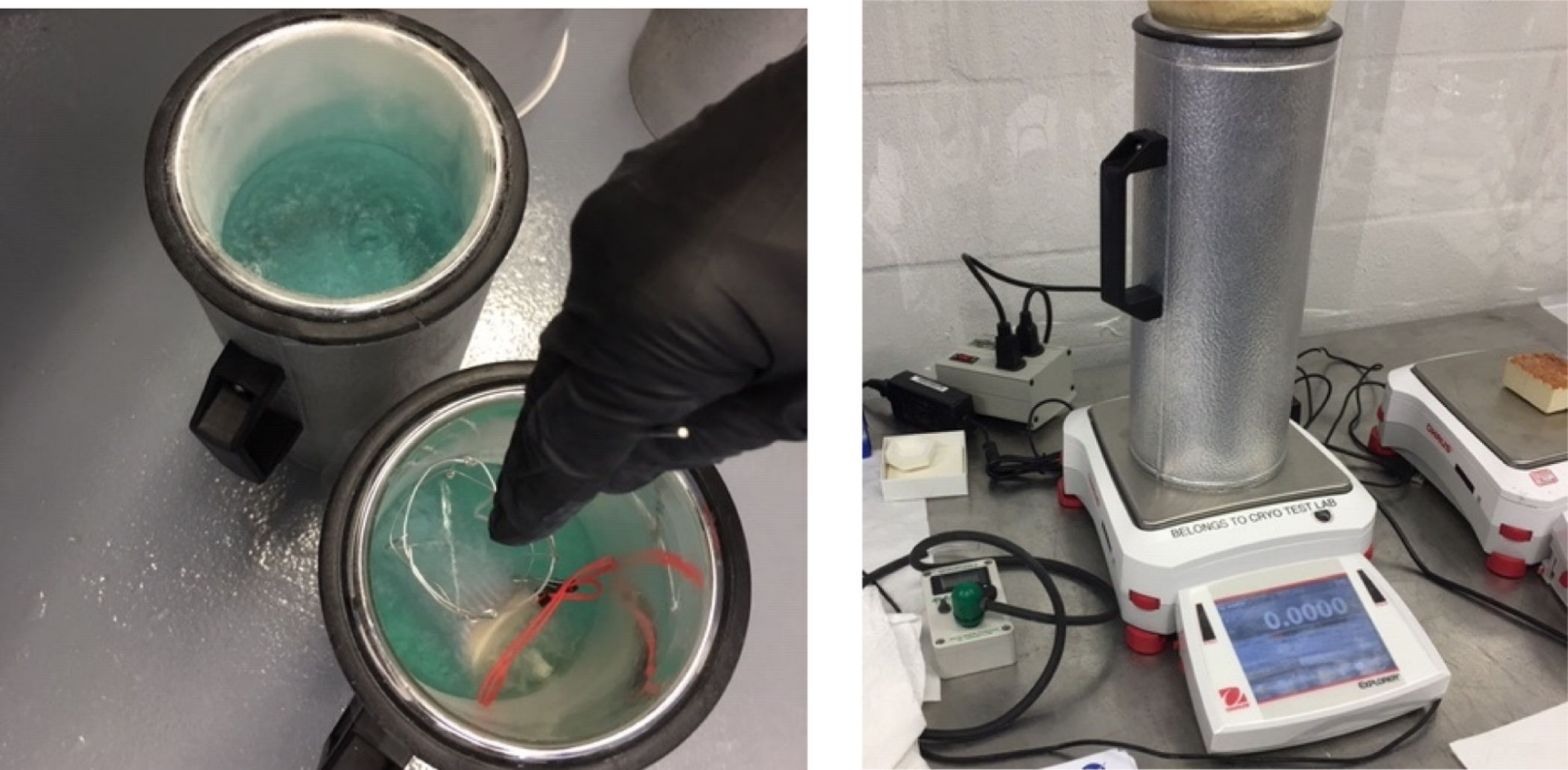
Prototype CFC core modules being charged in liquid oxygen (left) and placed in two-liter dewar flask on weight scale for long-duration burn-down test (right)

**Figure 6.**
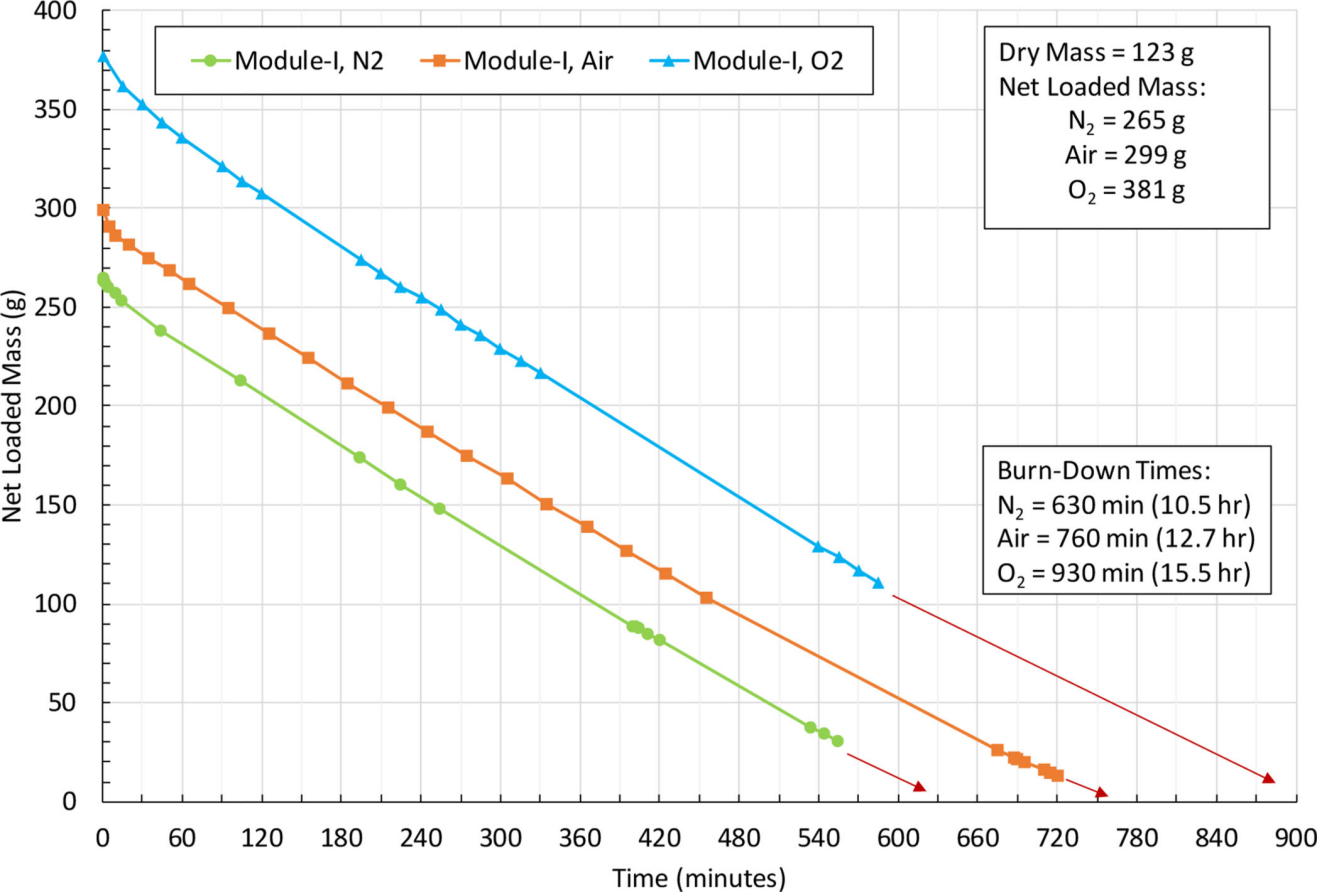
Net loaded mass burn-down profile: Module I, all fluids

Testing of three prototype CFC modules was performed using LN_2_, L-Air, and LO_2_ for both the cooling and the molecular charging of the units. The physical characteristics of three CFC core module test articles are given in table 1. A photograph of the three different modules is given in figure 4. In figure 5, a prototype CFC core module is shown being charged in liquid oxygen and placed in two-liter dewar flask on weight scale for long-duration burn-down test. Details of the tests of Module I are given for all three fluids is presented in figure 6. The desorption, or burn-down, test results for three CFC core module test articles are summarized in table 2. The performance for three prototype modules are summarized in table 9. These tests were performed using a container with a heat leakage rate of approximately 1.6 W. Modules I and III meet the basic requirement (one hour of breathing O_2_ after 9 hours of dormancy) for the rescue breathing pack application while Module II (the smallest size of the three) would need to be increased in size by about 50%. There are many options to consider for optimization and reserve capacity, but the key part of meeting the dormancy requirement is the container heat leak.

## 6. Future methods of charging/discharging

The primary design factors include physical configuration (spiral coil or parallel plate), the one hour O_2_ supply (mass), the O_2_ supply flow rate, the dormancy period (mass loss rate), the container heat leakage rate, the overall shape/weight, and the on-demand heat supply. Another important factor includes balancing the heat supply depending on breathing load variations. The overall ruggedness (impact resistance) of the module is expected to be excellent due to the very high mechanical energy-absorbing nature of the aerogel composites.

**Figure 7.**
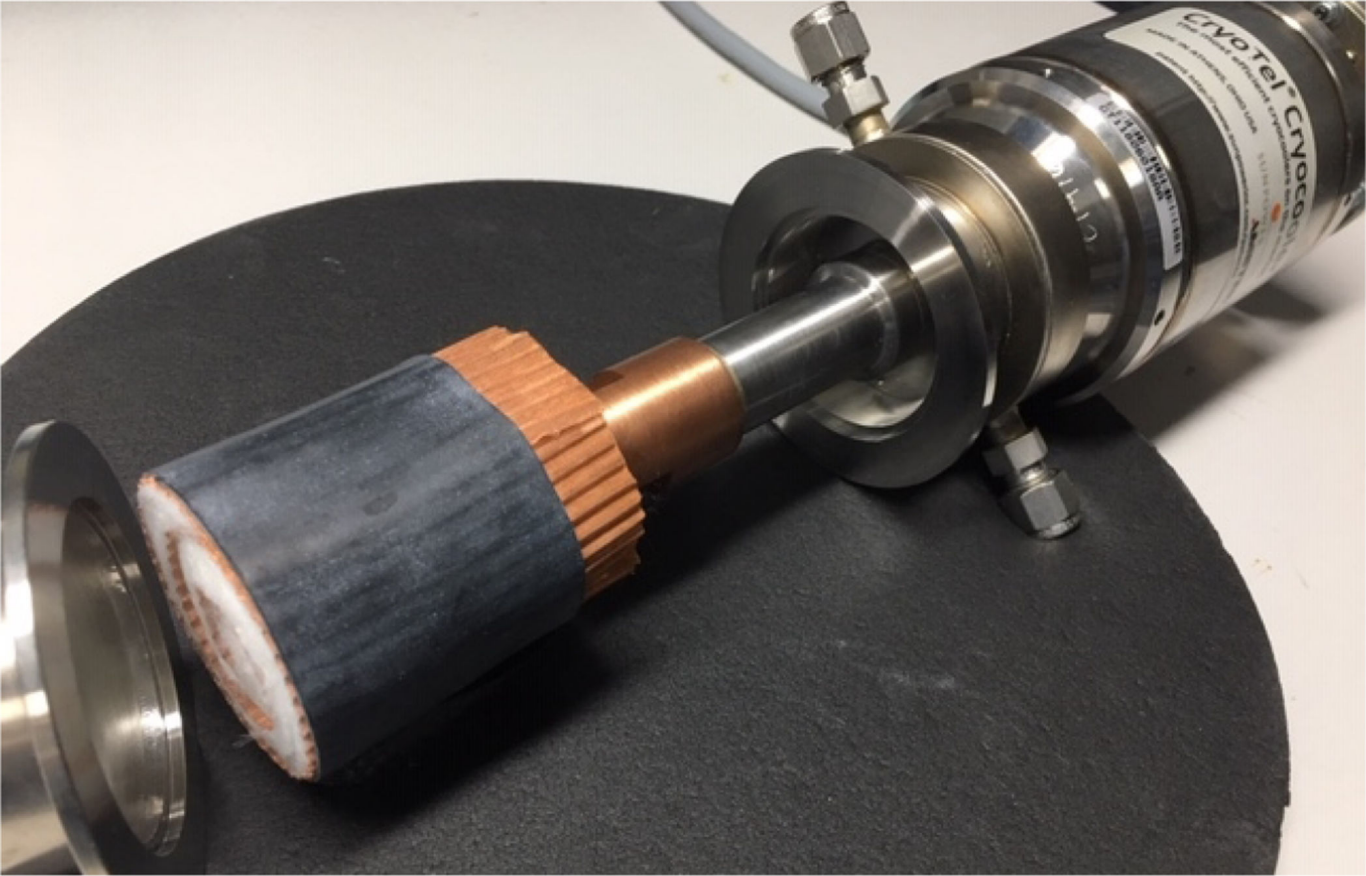
Notional concept of a future systems for liquid-free charging of CFC modules: the cryocooler (right) provides the refrigeration to a cryogenic temperature in parallel with supplying low pressure gaseous oxygen to module for molecular adsorption to the fully charged state.

Future or alternative systems for different methods of charging and discharging of the CFC core modules are also being investigated. Of particular interest is “liquid-free” charging. Charging by simple immersion in liquid or by liquid supply is quick and effective, but is not required. The concept for liquid-free charging is to thermally connect the CFC core to a cryocooler cold head, as suggested in figure 7, and then feed the process gas (gaseous oxygen in this case) to the module for adsorption at any below-ambient temperature according to the refrigeration capability of the cryocooler. One larger cryo-refrigerator unit, such as a pulse tube type, can be readily adapted for the simultaneous charging of multiple modules. Alternatively, a simple reservoir of liquid nitrogen (LN_2_) or circulation of LN_2_ through a heat exchanger could be used to conductivity cool the modules for concurrent supply of gaseous oxygen for adsorption and charging of the modules.

## 7. Conclusion

The technology of using the phenomena of cryo-adsorption afforded by the Cryogenic Flux Capacitor technique appears feasible for the breathing oxygen application. For the core modules tested, the liquid volume equivalent for oxygen is approximately 103% (that is, the solid-state molecular storage density is slightly higher than that of the liquid phase. The size and weight should be well within the current bounds. Promise is shown for additional features and capabilities such as CO_2_ sequestration and longer usage durations for a relatively smaller envelope/weight.

## Figures and Tables

**Table 1.** Physical characteristics of three CFC core module test articles.

Module	Description	Overall Dimensions	Dry Weight	Aerogel Weight

I	Plain coil w/ heater and lead wires	83-mm diameter x 10-mm long	124 g	60 g
II	G10 case w/ G10 tube (30 g)	50-mm diameter x 100-mm long	123 g	30 g
III	Black case w/ concentric fittings	100-mm diameter x 175-mm long	503 g	64.4

**Table 2.** Burn-down test results of three CFC core module test articles.

Module	Dry Mass	Fluid	Loaded Mass	Net Fluid Mass	Avg Burn-down rate*	Flow Rate of Gas	Burn-down Time*	Total Gas Volume	Inc. time vs. N_2_ baseline

	*g*		*g*	*g*	*g/min*	*SLM*	*hours*	*L @STP*	%
I	124	N_2_	387	265	0.42	0.34	10.5	214	-
		Air	423	299	0.39	0.32	12.7	243	21
		O_2_	501	377	0.41	0.29	15.5	267	48
II	123	N_2_	239	116	0.36	0.29	5.5	95	-
		Air	267	144	0.36	0.29	6.3	111	15
		O_2_	306	182	0.34	0.24	9.0	125	64
III	503	N_2_	1,145	642	0.51	0.41	21.0	514	-
		Air	1,273	769	0.53	0.43	24.3	631	16
		O_2_	1,466†	963†	0.52†	*0.36* †	*31.5* †	*688* †	*50* †

* Tested in pre-cooled two-liter dewar flask with foam cap (baseline flask heat leak of 1.6 W with LN2)

† Estimated based on test data from Modules I and II

**Table 3.** Performance of three CFC core module test articles in a two-liter dewar flask (1.6 W).

Module	Description	Volume	Dry Mass	Aerogel Mass	O_2_ Mass	Burn Rate	Burn Time	Time @116g O_2_ left

		cm^3^	g	g	g	g/min	hr	hr
I	Plain coil w/ heater and lead wires	261	124	60	381	0.41	15.5	9.5
II	G10 case w/ G10 tube (30 g)	157	123	30	182	0.34	9.0	3.7
III	Black case w/ concentric fittings	550	503	64	963	*0.50* *	*31.5* *	*28.0* *

* *Estimated*
